# Coupled experimental–computational study of Lapidus plates for the high-performance of surgical techniques for hallux hypermobility correction

**DOI:** 10.1186/s13018-026-06930-0

**Published:** 2026-05-11

**Authors:** Natali Mancera-Campos, L. Padrón-Cabrera, M. Llorens-Eizaguerri, J. A. Bea, A. Vidal-Lesso, J. Bayod-López

**Affiliations:** 1 Department of Mechanical Engineering, Universidad de Guanajuato, Salamanca, Mexico; 2 Department of Mechanical Engineering, Universidad de Zaragoza , Zaragoza, Spain; 3 Aragon Institute of Engineering (I3A), Universidad de Zaragoza, Zaragoza, Spain; 4 Applied Mechanics and Bioengineering Group (AMB), Aragon Institute of Engineering Research (I3A), Universidad de Zaragoza, Zaragoza, Spain; 5 Department of Traumatology and Orthopedics, Faculty of Medicine, Universidad de Zaragoza, Zaragoza, Spain; 6 Orthopedic surgery and traumatology, Hospital MAZ, Zaragoza, Spain

**Keywords:** Arthrodesis, Foot biomechanics, Cuneometatarsal fixation, Lapidus construction, Numerical analysis

## Abstract

**Background:**

For the correction of Hallux hypermobility through surgery, Lapidus plates have been studied as surgical fixation devices to evaluate the procedure’s success and its implications for biomechanical foot performance. However, significant knowledge gaps persist regarding the behavior of these devices and their biomechanical characteristics following implantation.

**Methods:**

This work studies two types of Lapidus plates through a coupled experimental-computational analysis. The first is a plantar fixation device, and the second is a helical device; both are used as fixation tools in Cuneometatarsal Arthrodesis. For this, mechanical tests specifically compression and fatigue are conducted with specimens of cadaveric feet implanted in the first cuneometatarsal joint (FCMJ) with each of the Lapidus plates. To the knowledge of this research team, for the first time reported in literature, complete foot specimens are used for the experimental tests. At the same time, numerical foot models have been developed to generate numerical data validated against experimental results. This approach enables the identification of additional structural information, through numerical methods, which is then complemented by electron microscopy analyses of the plates after experimental testing.

**Results:**

The highest micro-deformation was found in the first metatarsal of the foot with a helical plate ($\:{8.61\times\:10}^{-5}$ mm/mm) and in the fifth metatarsal of the foot with a plantar plate ($\:{5.25\times\:10}^{-5}$ mm/mm). Both numerical and experimental analyses agreed within 98%. A correction of Hallux hypermobility up to 82.28% was achieved with the Lapidus arthrodesis, and bone failure was recorded at forces of 425 N and 275 N at the helical and plantar insertions, respectively.

**Conclusions:**

The results suggest that the Lapidus plate’s helical configuration is superior, both structurally and functionally, to the plantar-only configuration, indicating that multi-planar fixation provides better mechanical stability than single-plane plantar fixation.

## Introduction

Hallux hypermobility is generally described as an excessive dorsiflexion movement of the head of the first metatarsal caused by an instability of the FCMJ [1–3]. This leads to abnormal movement of the first ray and its subsequent deformation, as well as to significantly altering the distribution of mechanical load throughout the foot [4–6]. Due to the above, hypermobility is pointed out as the first cause of various pathologies, among which Hallux Valgus (HV) development stands out [2, 4, 7–16], with a prevalence more significant than 35% in the elderly population of 65 years old [8–10] and 23% in the population between 18 and 65 years old [11, 12]. HV in severe stages considerably reduces people’s quality of life, and surgical treatment becomes essential to correct the position of the first ray and relieve the pain caused by this condition. There are various surgical treatments available to correct moderate to severe HV [8, 17–20]. Although orthopedic specialists indicate minimally invasive treatments in most cases [21, 22], arthrodesis is the most common treatment to correct this foot condition at advanced stages [23–27]. This procedure also offers a wide range of variations related to the fixation element used, among which we can mention the use of staples, screws, and Lapidus plates [25, 27–31]. Among these variations, a constant evolution remains evident in the search to improve postoperative results in patients treated with this procedure, as joint non-union is the complication most frequently reported in the literature [32] and rates reach up to 11% [10]. Using Lapidus plates as a fixation tool has gained relevance, as it has been shown that postoperative results using a Lapidus-type arthrodesis are superior to those obtained with other fixation alternatives [25, 28, 31, 33–38]. Although the variety of plates available is large, all of them can be grouped according to their fixation area, such as plantar, dorsal, medial, and dorso-plantar (spiral) [23, 39], thus generating a growing interest in the investigation of the success of fixation relative to the type of plate used, dividing opinions on the optimal position to place these devices [23, 24, 40, 41]. However, even with these investigations, much about the structural and biomechanical performance of Lapidus plates once implanted in humans remains unknown. Specifically, about the level of stress and strain reached, both in the plates and the bones of the joint to which they are attached. The biomechanical analyses documented in the literature have conducted experimental tests of various fixation methods for Lapidus plates on the bones of the FCMJ [24, 26, 42–45]. Most of these are tested in cadaveric specimens consisting only of the two main bones of the FCMJ (first metatarsus and medial cuneiform) [25, 28, 35, 41, 44–46] and in others, anatomical models are used, which usually include the four bones of the first ray (medial cuneiform, first metatarsus, first proximal phalanx and first distal phalanx) [26, 30, 42, 47, 48]. Kameel Garas et al. [29] and Ettinger et al. [43] have conducted biomechanical evaluations using complete foot specimens, comparing two types of constructions to fix the CMJ. Kameel Garas [29] compare the compression achieved with two constructions with Lapidus plates, one fixation and one compression, when an additional lag screw is used in each of these and when not—demonstrating with his work that the use of an additional lag screw in a Lapidus plate fixation provides a high initial level of compression that is maintained for an extended period, in contrast to the construction that only uses the plate alone. For their part, Ettinger et al. [43] performed a biomechanical evaluation to compare CMJ fusion with the use of crossing lag screws versus a fixation with a lag screw with a locking plate. Concluding that fixation with the construction that includes a Lapidus plate with a lag screw shows significantly superior results and greater stability than the use of only crossing lag screws. The information provided in both works can serve as a guide to help surgeons choose between the constructions compared. However, no work has yet reported information about the state of stress and deformation of the Lapidus plate and the foot after using any Lapidus-type construction that fixes the CMJ applied to complete foot specimens. Pasapula et al. [49] were the first to develop a numerical model of the foot to study stress concentrations resulting from two commonly used types of arthrodesis, employing contact fixation, instead of a fixation device, to replicate the technique behavior in the foot. Their work provided an overview of the postoperative structural behavior of the involved bones and the surrounding soft tissues, emphasizing the importance of evaluating the inner stresses of the joint in the presence of fixation elements. Therefore, this work aims to evaluate the structural performance of two types of Lapidus plates to identify the level of tension and deformation that these devices can experience after implantation in the foot and, consequently, obtain information about their expected lifespan. On the other hand, electron microscopy analysis will enable the study of these fixation plates once removed from the human body, allowing for the identification of potential failure points after they have been in active use, thereby providing information that is currently unknown. The above will also generate relevant information about the structural implications on the bones of the joint, allowing a better understanding of the biomechanical behavior of the entire foot after the surgical procedure.

## Materials and methods

### Experimental evaluation

**Fig. 1:**
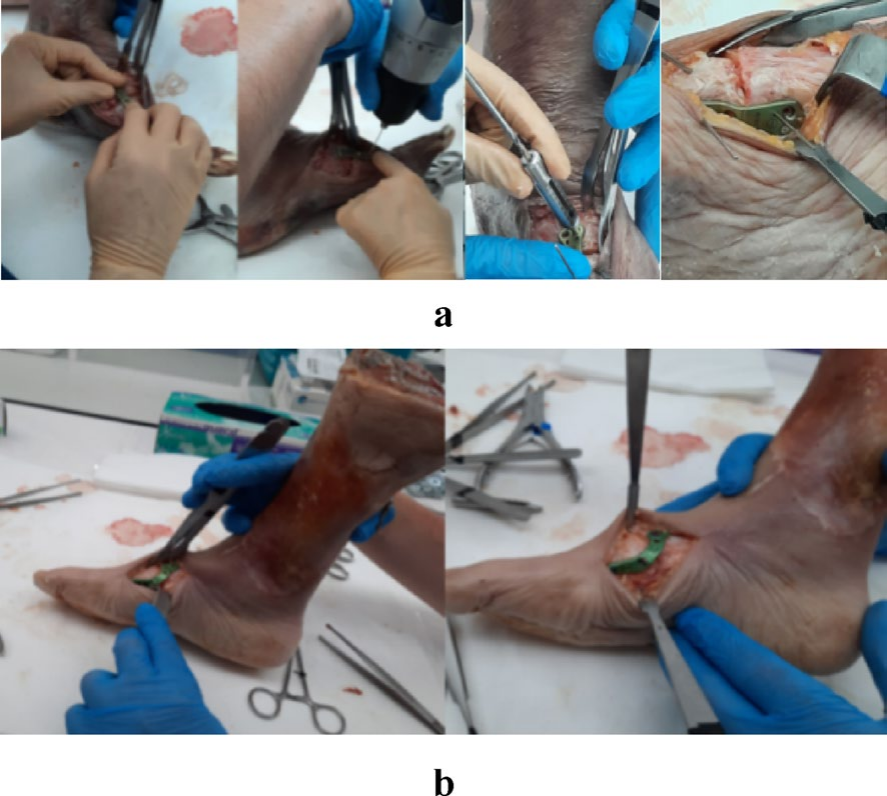
Surgical procedure used to insert the Lapidus plates:** a** plantar and** b** helical

The experimental evaluation consisted of studying the load-bearing capacity of two types of Lapidus plates (plantar and dorsomedial) inserted into two cadaveric foot specimens with no apparent disease. This study aimed to assess the structural effects on the bony elements of each foot sample tested, as well as on the Lapidus plates. It is essential to note that all methods employed in this work adhere to the guidelines outlined in the Declaration of Helsinki, as adopted by the World Medical Assembly (WMA). The foot cadaveric specimens had no pathological conditions or injuries, indicating no hypermobility in the first cuneometatarsal joint. Therefore, to simulate hallux hypermobility, an orthopedist induced a luxation between the medial cuneiform and the first metatarsal in each sample, dislocating both bones and producing maximal hypermobility. This resulted in a cuneometatarsal angle within the range reported for preoperative cases [50, 51] and an injury type A2 according to Mehlhorn et al. [52] (see Fig. 1). This step ensured that the Lapidus plates analyzed could stabilize the joint and experience excess mobility, as in the pathology. Figure 1a–b shows the procedure used to generate a joint dislocation in one specimen.

**Fig. 2:**
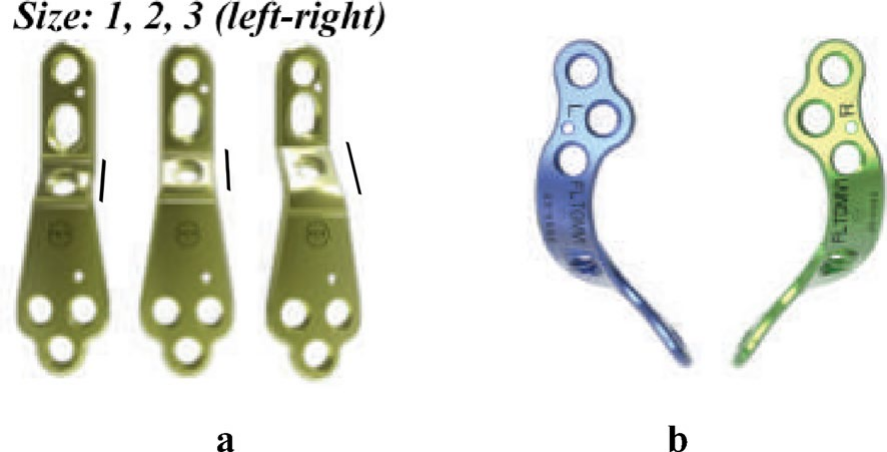
Plates used for load tests: **a** plantar Lapidus plate and **b** helical [53]

Two different plates were evaluated, a plantar Lapidus plate and a helical (helical) plate (see Fig. 2a–b). Both designed and produced by the medical device company New Clip Technics and indicated for use in surgical treatment (cuneometatarsal arthroplasty) for hypermobility of the first metatarsocuneiform joint and its associated pathologies, such as hallux valgus, osteoarthritis, functional deformity, and flat feet.

#### Compression tests

**Fig. 3:**
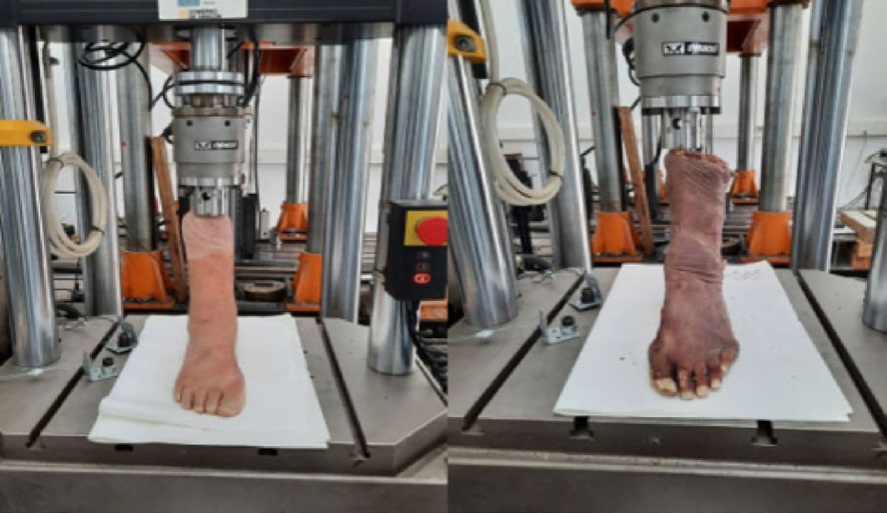
Installation of the cadaveric specimens on the INSTRON machine

After implementing the surgical technique on the test specimens, compression tests were performed. Two feet were implanted, each one with a different Lapidus plate, previously described (see Fig. 2). For the tests, an INSTRON 8874 biaxial compression machine (Fig. 3) was used.

The Bluehill software version 2.17.649 was used to manage test data, and the test conditions were established as axial compression tests with data capture at a 0.01 s acquisition period. A ramp speed of 0.5 mm/s was established with extension displacement control. The end of the test was established by detecting 80 mm of extension as the end of travel. The test was stopped when the predefined extension level was reached.

The protocol described was applied to each test specimen until failure. Load-extension graphs were obtained from these tests, along with information on the fracture zones, once the maximum force that the tested specimens could withstand was reached. Figure 3 shows how the cadaveric specimens were secured in the INSTRON testing machine for axial compression tests.

#### Cyclical load tests

Cyclic load tests were performed on two standing cadaveric specimens, one for each analyzed plate. The plate installation process was the same as previously described (see Fig. 1a and b).

**Fig. 4:**
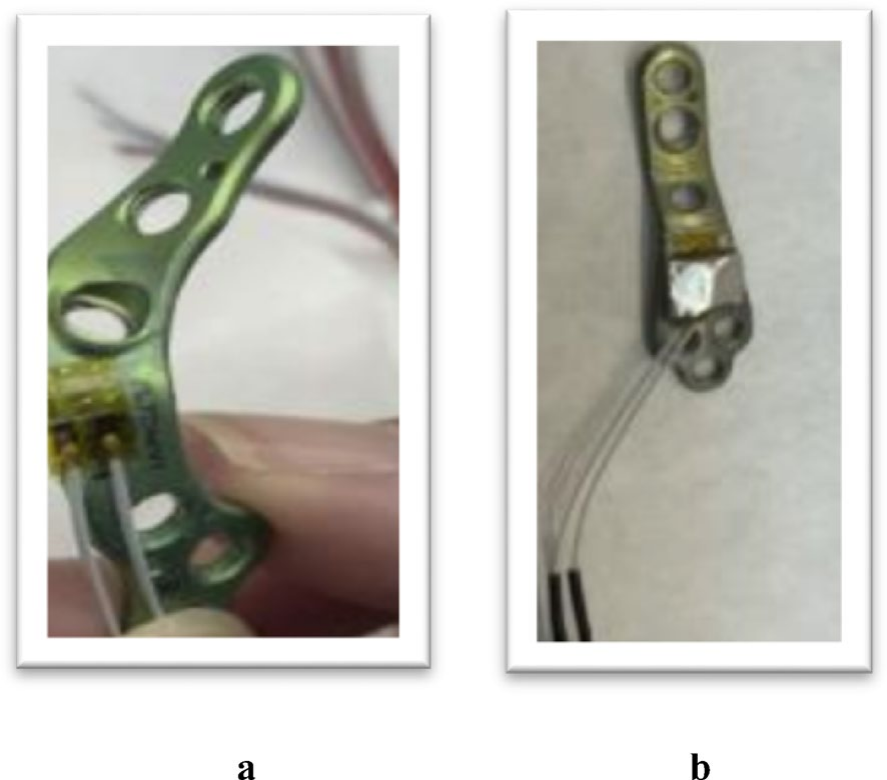
Instrumentation process using strain gauges. **a** plantar plate and **b** helical plate

For these tests, each plate was instrumented with strain gauges on both the foot specimen and the plate itself. The gauges used for this purpose were HBM, which has a resistance of 120 Ω ± 1.00%. Figure 4a–b shows the instrumentation process for the plates using strain gauges.

**Fig. 5:**
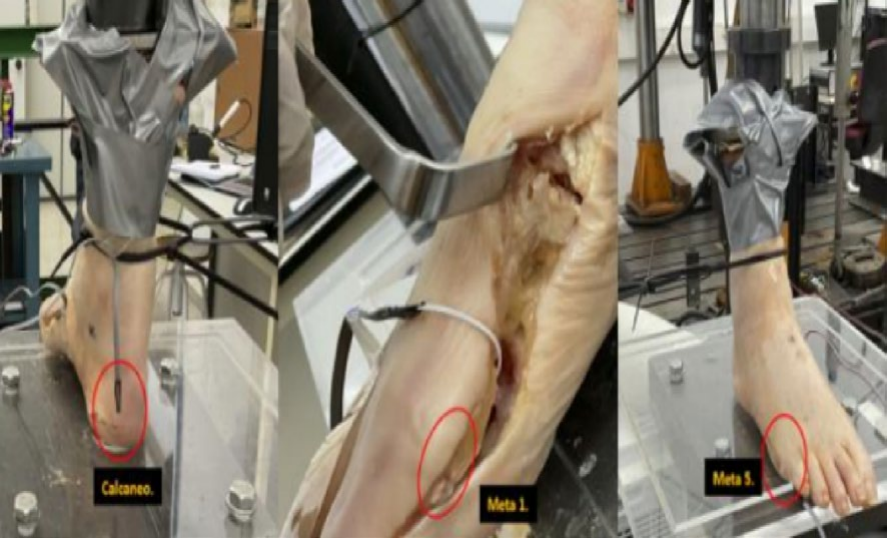
Fixation areas for the gauges on the pedal tripod

As part of the instrumentation process and to ensure proper adhesion of the gauges to the surfaces of the Lapidus plates, an acetone- and isopropanol-based spray cleaner was used. Afterwards, once the surface was clean, a Z70 fast-curing cold-curing adhesive (cyanoacrylate) from HBM was applied. In addition to the gauges placed on the plates, three more of them were added for each specimen (of the same brand and characteristics as those placed on the plates) at three strategic points on the plantar tripod. Four gauges were placed for each specimen: three on the bones and one on the plate. After each of the foot specimens was instrumented correctly, the test conditions for the cyclic tests were established. The posture defined for performing the tests (both cyclic and compression) was the phase of unilateral medial stance, corresponding to the second rocker of the gait cycle. The strain gauges were placed as follows: Gauge 1 on the inferior surface of the calcaneus; Gauge 2 below the base of the first metatarsal; and Gauge 3 below the base of the fifth metatarsal. Gauge 4, on the Lapidus plate (plantar or helical, depending on the specimen). Figure 5 shows the different views of the specimens, from which it is possible to identify each of the external points where the gauges were placed. Strain gauge data acquisition was performed using a KYOWA PDC-400 A system.

**Fig. 6:**
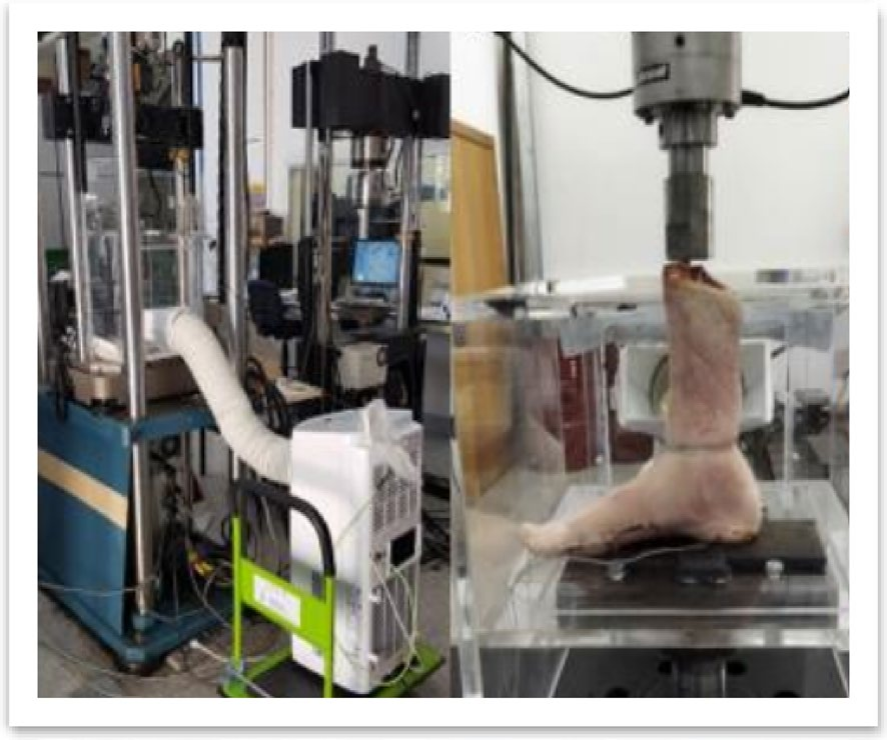
Fatigue testing machine, and a specimen standing in the analysis position

The cyclic loading tests were developed as follows. First, a cubic methacrylate urn was designed and constructed, with the upper surface open to access the tibia through the load cell (see Fig. 6). Subsequently, the urn was located in the INSTRON machine where the tests were performed with a dynamic rating of ± 5 kN and equipped with a 9000 BTU air conditioning system capable of supplying air within a range of 0°, – 1 °, and –  2 ° C to ensure the specimens’ preservation.

Following this, an initial axial compression force was set between 50 and 60 N, and starting from this, a 5-s axial compression ramp was applied, with the final bridge at the mean value of the sinusoid specific to each test. A total of 100,500 cycles were established to complete the test. Moreover, the second rocker gait position was established as the position to be analyzed, i.e., between the heel strike or first rocker and the toe-off phase of the Hallux. KYOWA’s exclusive DCS-100 A Series software, Version 04.50, was used for dynamic control.

### Evaluation of Lapidus plates with electron microscopy

Once the tests were completed, the Lapidus plates were removed from the standing cadaveric specimens on which they were installed. Then, they were cleaned to remove any impurities from the biological tissues and from the treatment they underwent during instrumentation with strain gauges. Once the plates were cleaned, each plate was analyzed by electron microscopy. This analysis was performed using a Carl Zeiss MERLIN™ field emission scanning electron microscope (FESEM).

**Fig. 7:**
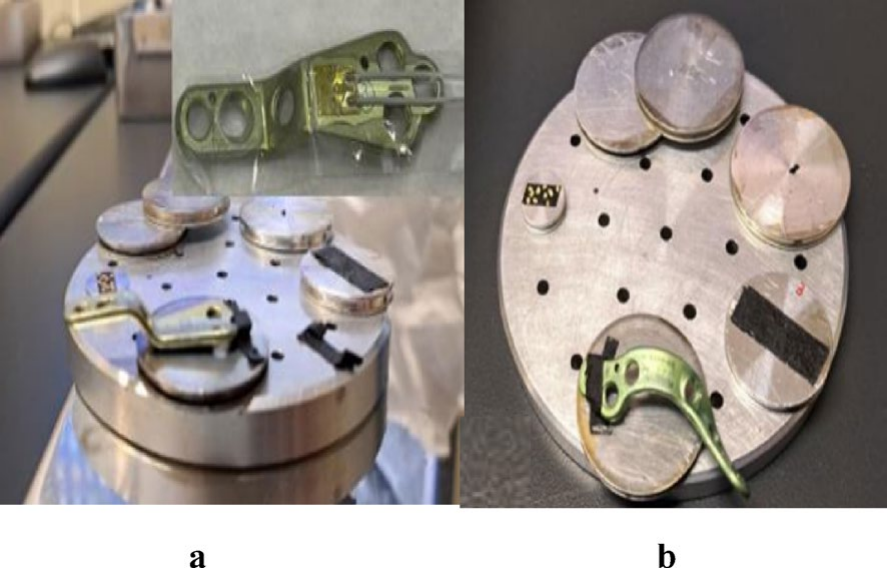
Scanning electron microscopy. **a** helical plate, and **b** helical plate

The plates were analyzed individually, starting with the helical plate and ending with the plantar plate. The general procedure involved mounting the plates on a microscope slide and adjusting their position to ensure the surfaces were adequately evaluated. The surfaces of interest were then evaluated for microcracks or signs of them that could have been induced by the loading tests performed on the implanted feet. Figure 7a–b show the helical and plantar plates mounted on the slide, respectively.

### Comparison of experimental methodology with literature

Table 1 presents a direct comparison of experimental testing methodologies reported in literature with those reported in this work. It is possible to appreciate how, although each of the analyzed specimens differ in the conditions under which it has been evaluated, the use of complete foot specimens can allow a more accurate prediction of the failure force by considering the interaction between all the parts that make up the complete structure of the foot.

**Table 1:** Comparison of the different methodologies used in the literature with the one used in this work

References	Specimens tested	Testing method	Plate position	Failure load	Numerical evaluation
This work	Complete fresh-frozen human feet	Compression and cyclic tests with a detecting extension of 80 mm and a dynamic rating of ± 5 kN, respectively. 100,500 loading cycles were defined	Plantar locking plate and helical locking plate	Plantar plate failure load: 275 N. Helical plate failure load: 425 N	A numerical foot model was developed and validated with experimental results. Structural parameters such as Strain, Stress, and displacement were analyzed
[25]	Matched pairs of fresh-frozen human bones, first cuneiform and first metatarsal	Cyclic test with an amplitude load of 31 N in a plantar-to-dorsal direction. 1000 loading cycles were defined	Medial locking plate	Failure load of 162.9 N	NA
[26]	Composite synthetic bone pairs, first cuneiform and first metatarsal	Compression and cyclic tests with an amplitude load of 51 N in a plantar-to-dorsal direction. 5000 loading cycles were defined	Medial locking plate and plantar locking plate	Medial plate failure load: 324 N. Plantar plate failure load: 377 N	NA
[44]	Composite bone pairs, first metatarsal and medial cuneiform	Cyclic test with a preload of 25 N in the planar surface. 30 initial loading cycles until fail	Medial, dorsal and plantar plate fixations	Medial plate failure load: 260.4 N. Dorsal plate failure load: 285.03 N. Plantar plate failure load: 351.9 N	NA
[48]	Matched pairs of cadaveric human bones, first cuneiform and first metatarsal	Bending and compression tests with an initial displacement of 5 mm	X locking plate	Maximum force value of 70 N	NA
[54]	Pairs of fresh-frozen human bones, first cuneiform and first metatarsal	Cyclic test with an amplitude load of 40 N. 5000 loading cycles were evaluated	Plantar locking plate and helical locking plate	Plantar plate failure load: 225 N. Helical plate failure load: 210 N	NA
[55]	Pairs of fresh-frozen human bones, first cuneiform and first metatarsal	Cyclic test with a forces of 25–50 N, 50–150 N, 100–200 N and 200–300 N. Four increments of 50 cycles were evaluated	U-shaped plate and straight shaped plate	U-shaped plate failure load: 446.6 N. Straight shaped plate failure load: 540.6 N	NA

### Numerical analysis

The geometries of bones and tissues in this work were digitized through the scanning of a 49-year-old male volunteer with a height of 170 cm and a weight of 70 kg. Two different types of tomographies were used to capture the geometric details of all tissues in the foot [56]. The assembly of the bone structure and selected soft tissues was performed using the CAD software SolidWorks^®^. The physiological angle of the first metatarsophalangeal joint and Costa Bartani were measured to ensure that the values obtained from the constructed model were within the ranges considered normal, corresponding to those found in a healthy foot in a quiet stance position. The healthy foot model included a total of 28 cortical and trabecular bones, 61 articular cartilage segments; and 12 soft tissues, central plantar fascia (CPF), lateral plantar fascia (LPF), hallucis muscle (AH), abductor digiti minimi (ADM), flexor digitorum brevis muscle (MFDB), flexor digitorum brevis tendon (TFDB), extensor hallucis longus (EHL), flexor digitorum longus (FDL), flexor hallucis longus (FHL), tibialis anterior (TA), extensor digitorum brevis (EDB), and tibialis posterior (TP), selected as the most relevant due to their direct participation in the stability of the plantar arch and the flexion and extension function of the toes [56, 57].

Two platform segments were included to simulate the contact of the bony structure with the plantar soft tissue. The foot model discretization yielded a mesh comprising 1,582,868 tetrahedral and hexahedral elements. This was selected through a previous convergence analysis, which also enabled the determination of the optimal mesh size to reduce the analysis computation time. The joint interaction was defined as the surface-to-surface interaction between cartilage structures, allowing for free movement between them. On the other hand, the inner surfaces of the cartilages were joined to the surface of their corresponding bones, with contacts that restricted the movement between them in all directions. The contacts between bones and soft tissues were defined by associating the corresponding insertion points through constraint equations. Using the numerical model of the healthy foot as reference [58], some modifications were made to it to generate a second model with the characteristics of a pathological foot so that, under the action of body weight in mid-stance gait position, it would reproduce hypermobility of the cuneometatarsal joint, modifying the values of the physiological angles measured initially. The hypermobility of the area was achieved, reducing the pinball radius of the cuneometatarsal joint contacts by 80% compared to the original model. As mentioned before, the effect of the peroneus longus tendon force was determined by applying two loads of 17 N [49] each to the respective bones of the CMJ.

#### Arthrodesis numerical models

**Fig. 8:**
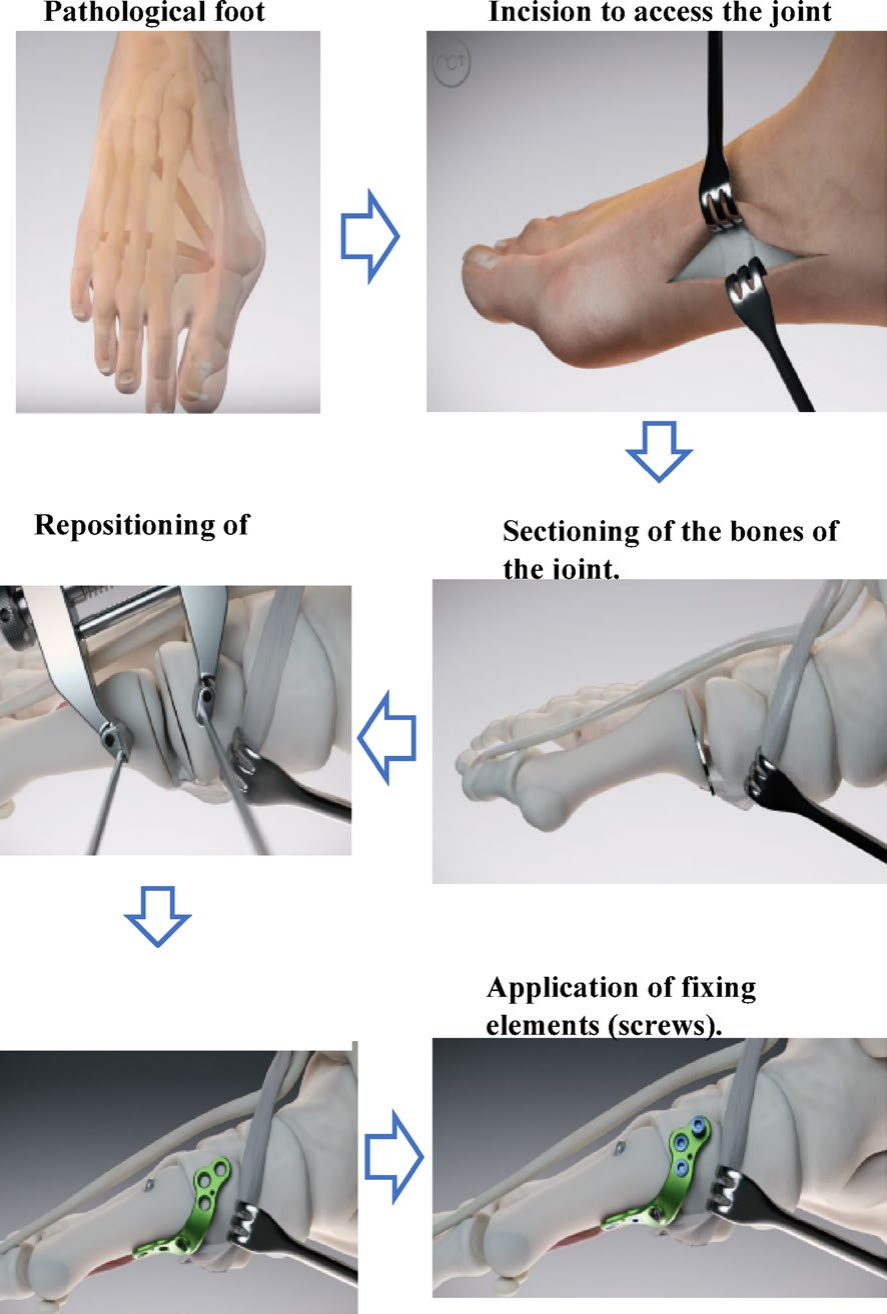
Steps followed to reproduce the surgical technique on the different arthrodesis models [53]

Once the pathological behavior of the developed foot model was verified, it was employed to reproduce the Lapidus-type arthrodesis using two different fixation tools—a plantar Lapidus plate and a helical Lapidus plate. The insertion of both fixation tools was performed according to the methodology shown in Fig. 8, as provided by New Clip Technics, the company that manufactures these medical devices. All these procedures were implemented in the ANSYS design module.

**Fig. 9:**
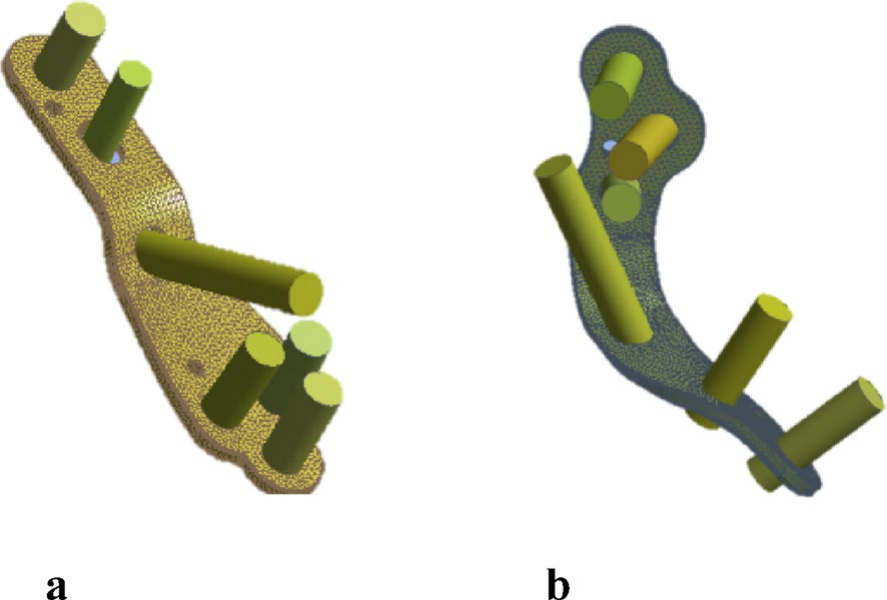
Screws for fixation system of the Lapidus plates: **a** plantar plate and **b** helical

The plantar and helical plates analyzed in this work were scanned in a similar way to foot specimens. Figure 9 shows the scanned fixation tools with their construction fixation.

**Fig. 10:**
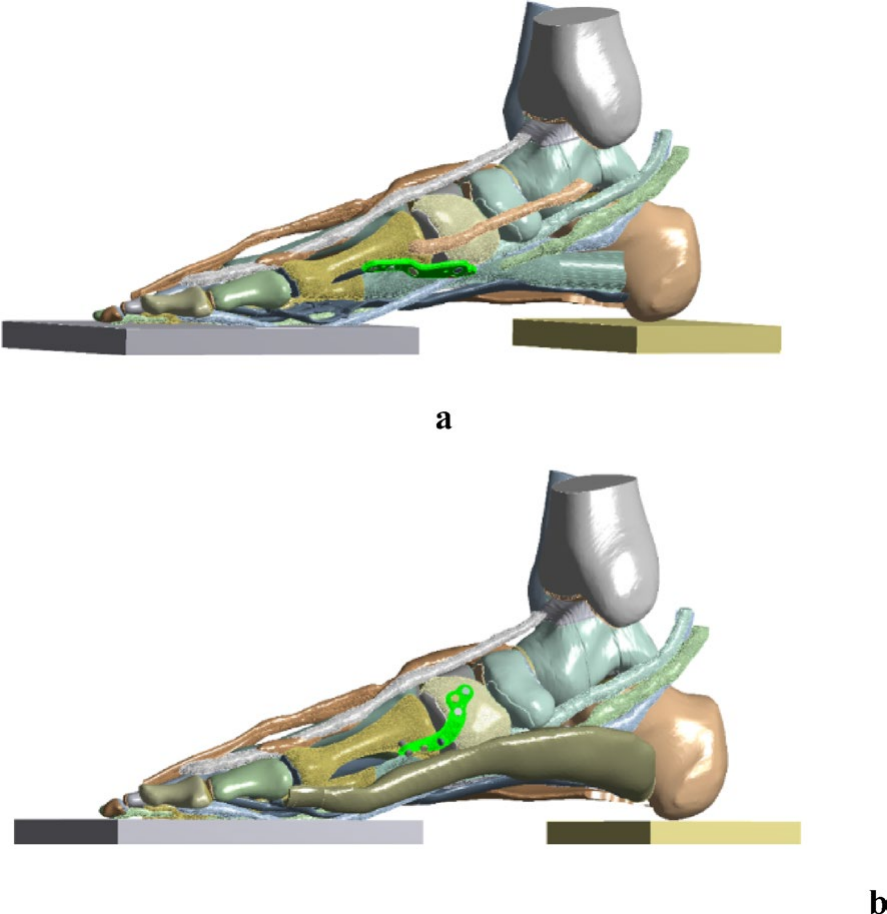
3D foot model with a Lapidus arthroplasty. **a** Plantar plate and **b** helical

Figure 10 shows the numerical models that represent the surgical procedure on the foot using the different scanned fixation elements.

#### Materials models

The material models used to simulate the behavior of each part of the foot were elastic, linear, and isotropic. This behavior was defined for bones, cartilage, and soft tissue. Although each of the tissues in the model exhibits physical non-linear behavior, the linear elastic models used here can be considered a good representation of the behavior at the strain levels managed [58]. The constants for each were obtained from the literature [58–66] and are reported in Table 2. For the Lapidus plate and screws used in the model in which the surgical technique was implemented, an elastic material model was also used, linear, and isotropic material model, whose properties were obtained from the literature too [67], and correspond to those of Titanium grade 5, the material with which this type of plate is manufactured.

**Table 2:** Constants of the material models which were defined for each part of the numerical foot model

Part of the numerical model	Material model constants
Cortical bone [66]	$\:E=17\:GPa\:,\:v=0.3$
Trabecular bone [56]	$\:E=0.7\:GPa\:,\:v=0.3$
Cartilage [61]	$\:E=10\:MPa\:,\:v=0.4$
AH, ADM, MFDB [60, 62]	$\:E=0.5\:GPa\:,\:v=0.47$
CPF [59, 63]	$\:E=1\:GPa\:,\:v=0.4$
LPF [59, 63]	$\:E=2.1\:GPa\:,\:v=0.4$
EDB [56]	$\:E=0.3\:GPa\:,\:v=0.4$
EHL, FHL [56]	$\:E=0.45\:GPa\:,\:v=0.4$
TFDB [56]	$\:E=0.5\:GPa\:,\:v=0.4$
FDL [56]	$\:E=0.34\:GPa\:,\:v=0.4$
TA [56]	$\:E=0.17\:GPa\:,\:v=0.4$
TP [56]	$\:E=0.19\:GPa\:,\:v=0.4$
Plantar soft tissue *(platform)* [68]	$\:E=54\:MPa\:,\:v=0.3$
Grade 5 titanium [67]	$\:E=114\:GPa,\:v=0.32$

#### Boundary conditions

**Fig. 11:**
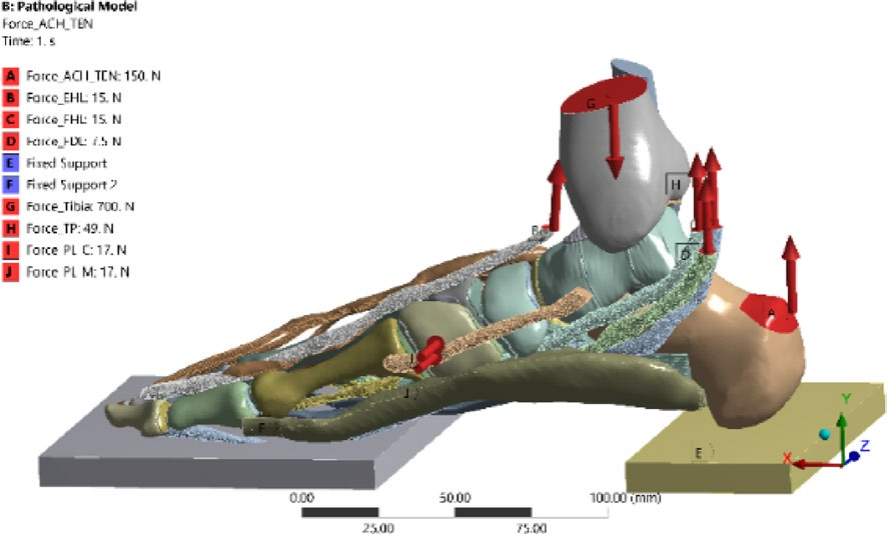
Forces exerted by the muscles through the tendons attached to the model

The analysis of the model was performed at mid-stance position under a load of 700 N applied vertically to the transverse surface of the tibial segment. The effect that each one of the muscles exerts through the respective inserted tendons was also considered, for which vertical forces were applied on the transversal surface of the TP, EHL, FHL, and FDL with magnitudes of 49, 15, 15, and 7.5 N, respectively [49, 64]. Since the geometries of the Achilles tendon and peroneus longus tendon were not included in the model, their actions were represented solely by forces applied to their insertion surfaces. For simplicity of the model, the action of the peroneus longus tendon was transferred to surfaces belonging to the middle cuneiform and metatarsal bones on which they have its insertion. The lower surface of the rectangular platforms was fixed in the plantar area to prevent sliding and to provide greater stability for the model. Figure 11 shows each of the forces mentioned. To provide stability to specific model components, movement constraints were defined to allow movement only in the Y direction of the tibia, fibula, and the transverse surfaces of the tendons on which the forces were applied. No Separation contact was also established between the upper surfaces of the platforms and the bones in connection with it, to allow interaction between both.

The material models and boundary conditions of the healthy foot model and the pathological foot model were the same; the only variation was established in the contacts of the cuneometatarsal joint to simulate the laxity of the area.

#### Arthrodesis model validation

The foot model validation was carried out by evaluating the load distribution across the foot. This was achieved by considering the contact forces in the hindfoot and forefoot regions of the pathological model developed in this study and subsequently comparing them with values reported in the literature and obtained from baropodometric assessments performed on a group of patients with conditions associated with hallux hypermobility. These values are reported in Table 3.

**Table 3:** Load distribution (BW%) in the forefoot (toes 1–5) and hindfoot (calcaneus)

Area	BW %	BW %
	Gutteck et al. [6]	This work
Calcaneus	68	72.74
Toes 1–5	16.3	20.21

**Fig. 12:**
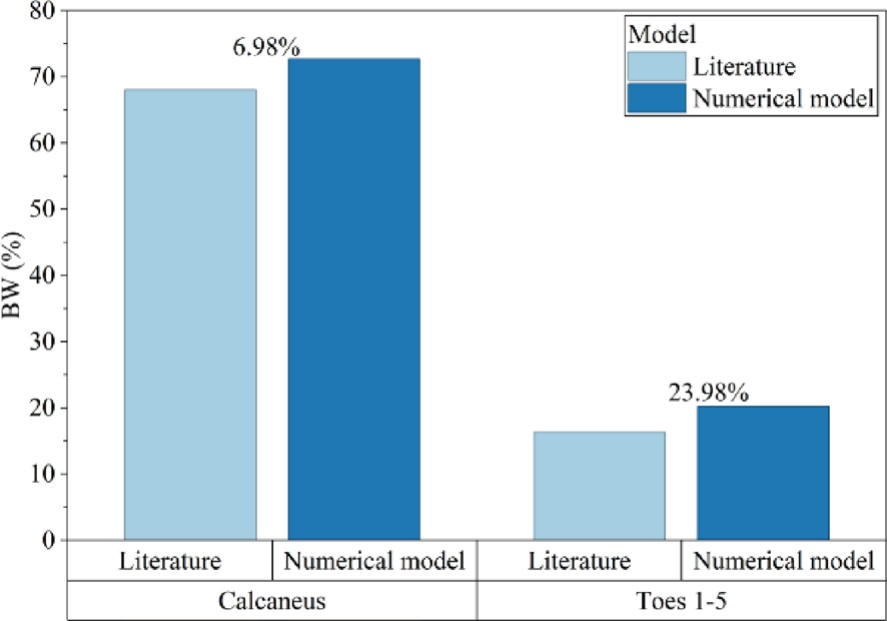
Percentage of load distribution in a hypermobile foot

The data in Table 3 show a greater variation in load distribution (of the model developed in this study compared to that reported in the literature) in the forefoot area (21.42%), with less variation in the hindfoot (6.73%). This variation is attributable to differences in evaluation methods, as the data reported in the literature were obtained from direct patient measurements. The boundary and contact conditions applied to the numerical model of the pathological foot are considered a second factor influencing these differences. However, the percentage difference is acceptable and suggests that the model developed in this study reflects the expected characteristics of a hypermobile foot. The percentage differences between the load distribution reported in the literature and those obtained from the numerical model are shown graphically in Fig. 12.

An evaluation of load distribution on both models, plantar and helical, is compared with values reported in the literature; the values of percentage difference are presented in Table 4. These values were obtained through baropodometric assessment performed on a group of postoperative patients who underwent corrective treatments for hallux hypermobility.

**Table 4:** Load distribution values ​​for the forefoot and hindfoot for each case evaluated in this work, and comparison of them with the data reported in the literature [6]

Area	BW %	BW % plantar	BW % helical
	Guttec et al.	This work (% of difference)	
Calcaneus	69.4	73.17 (5.28)	72.3 (4.09)
Toes 1–5	18.7	22.73 (19.45)	21.41 (13.51)

The percentage differences between the various analyses in the anterior foot region, 19.45% and 13.51%, reported in Table 1, while notable, are not considered to significantly impact the stress predictions made in this study. This is due to differences in the nature of the studies being compared. The reported load distribution percentages in the literature were derived from a group of patients who underwent various surgical techniques, including arthrodesis. Besides, the Lapidus plates studied in this work do not directly correspond to those used in the study by Gutteck et al.; however, the fixation conditions achieved by the surgical technique evaluated here allow comparisons with the literature to validate the results.

## Results

### Comparison between experimental and numerical results

**Fig. 13:**
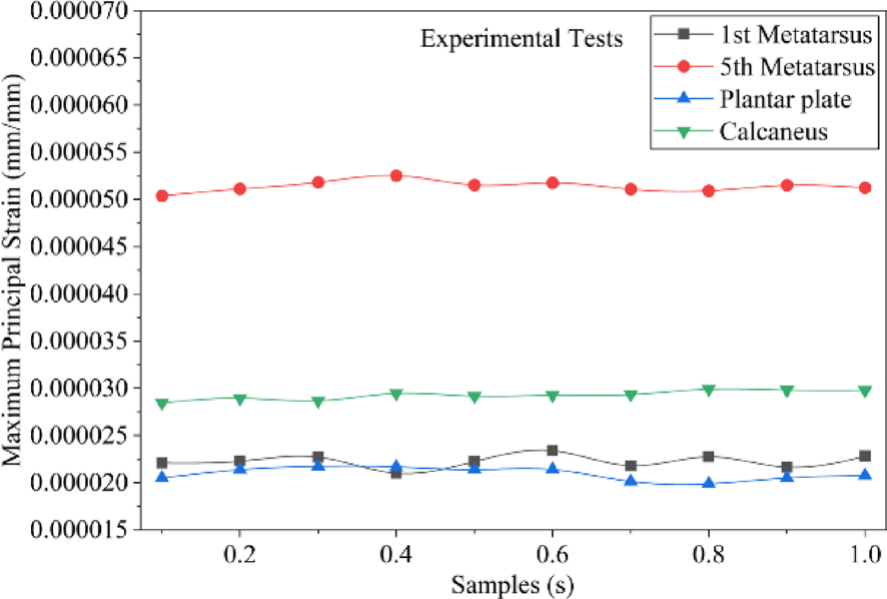
Principal strain behavior obtained in experimental tests for the plantar plate

**Fig. 14:**
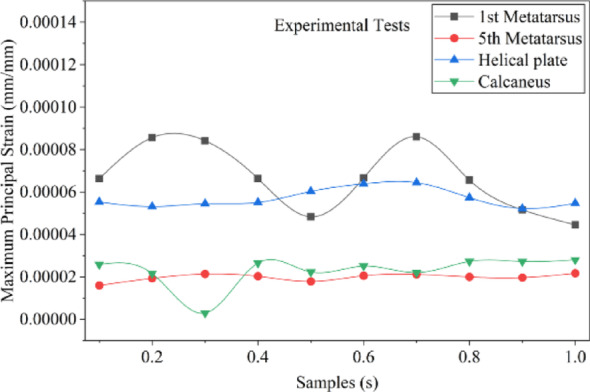
Principal strain behavior obtained in experimental tests for the helical plate

Through experimental evaluation using strain gauge instrumentation, it was possible to obtain micrometric deformation values in different areas of the foot and the Lapidus plates. The values obtained from both analyzed plates are shown in Figs. 13 and 14 for plantar and helical Lapidus plates, respectively.

It is essential to mention that the values shown in these figures are taken from the first second of the cyclical experimental tests.

**Fig. 15:**
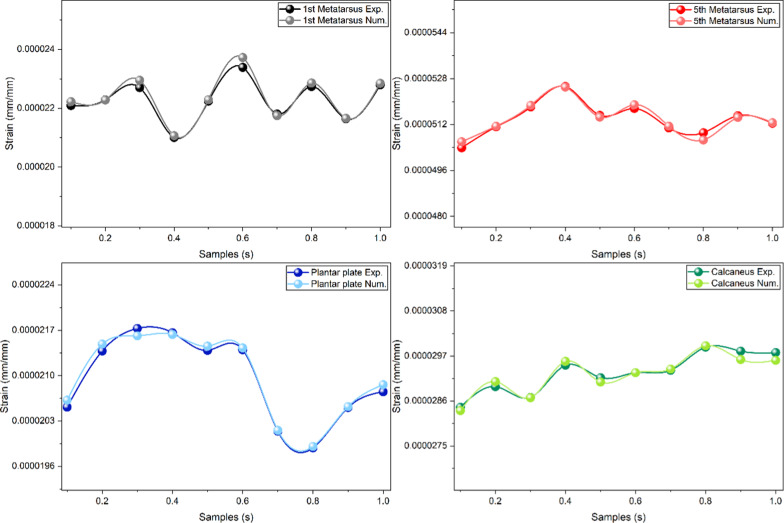
Principal strain behavior obtained in numerical tests for the plantar plate compared with experimental data

**Fig. 16:**
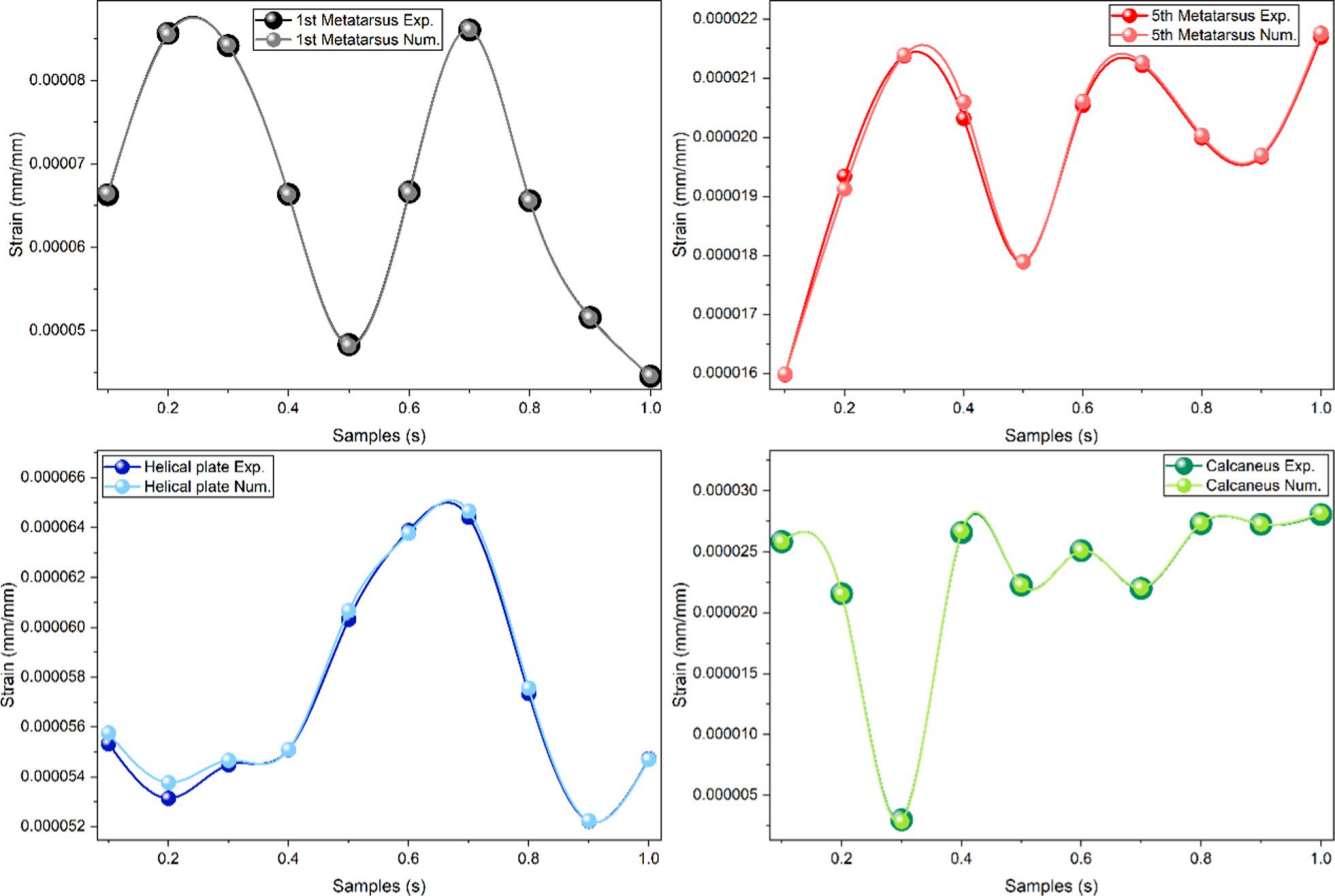
Principal strain behavior obtained in numerical tests for the helical plate compared with experimental data

Using the data obtained through experimental tests, a comparison was made between these values and those obtained from each numerical model, evaluating the same areas that were monitored with strain gauges during the experimental tests: the lower distal surface of the first and fifth metatarsal; the lower surface of the calcaneus; and the plantar and medial outer surfaces of the corresponding Lapidus plates (plantar and helical). Ten principal unit strain values were taken from each of the mentioned areas, which agree with the period time monitored from experimental tests. The values taken from each numerical model of the foot with arthrodesis compared with experimental data are shown in Figs. 15 and 16, for the plantar and helical cases, respectively.

### Evaluation of surgical procedures and the degree of correction of the pathology

**Fig. 17:**
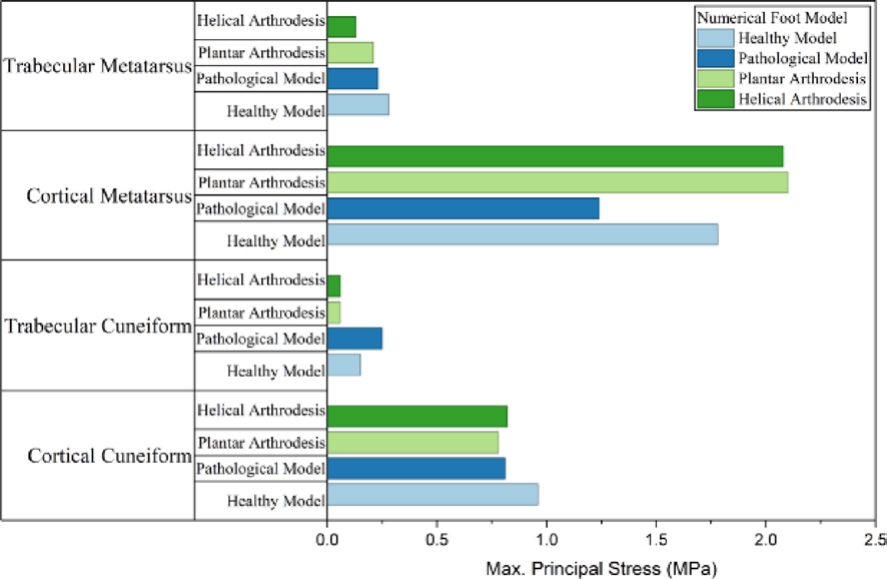
Comparison between models of stress values located in the different bone layers of the FCMJ

**Fig. 18:**
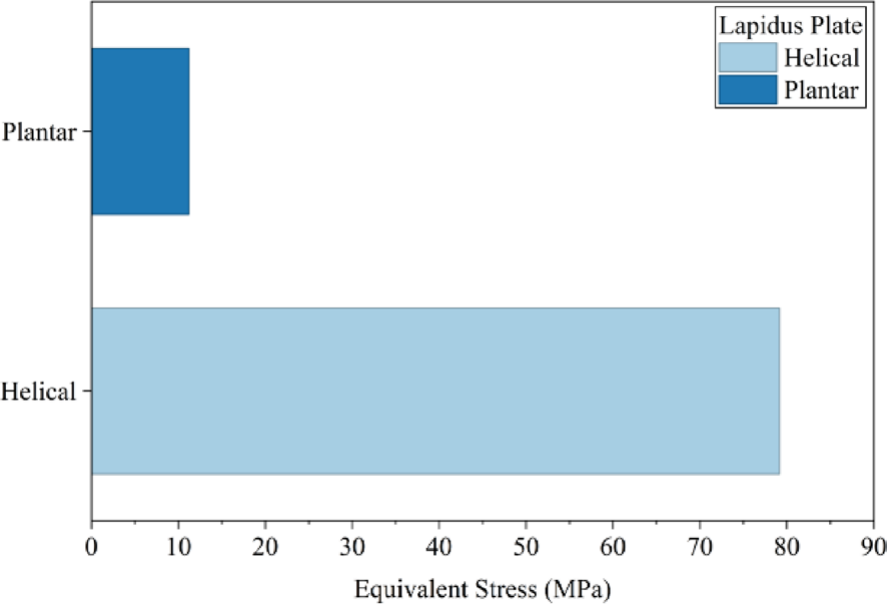
Comparison of the equivalent stress value between Lapidus plates

**Fig. 19:**
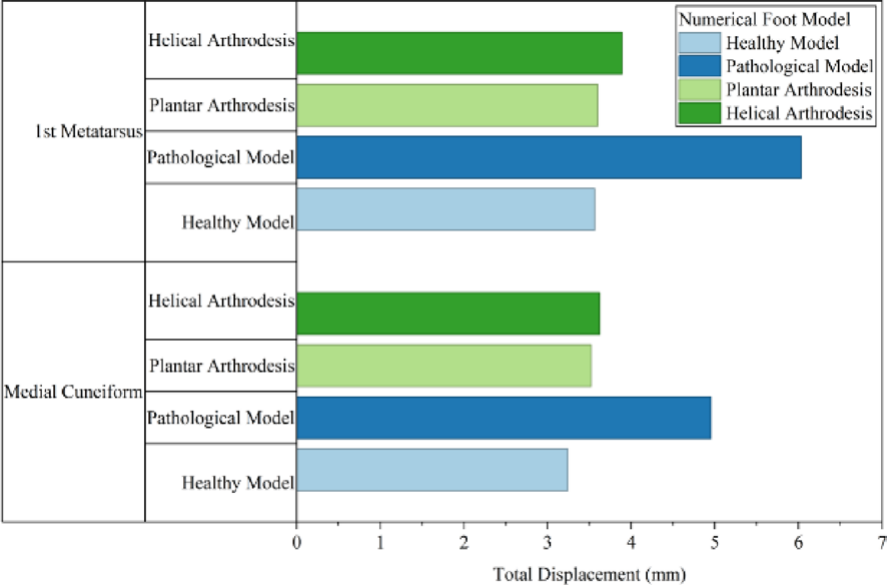
Total displacement values of the CMJ bones in the different foot models

**Fig. 20:**
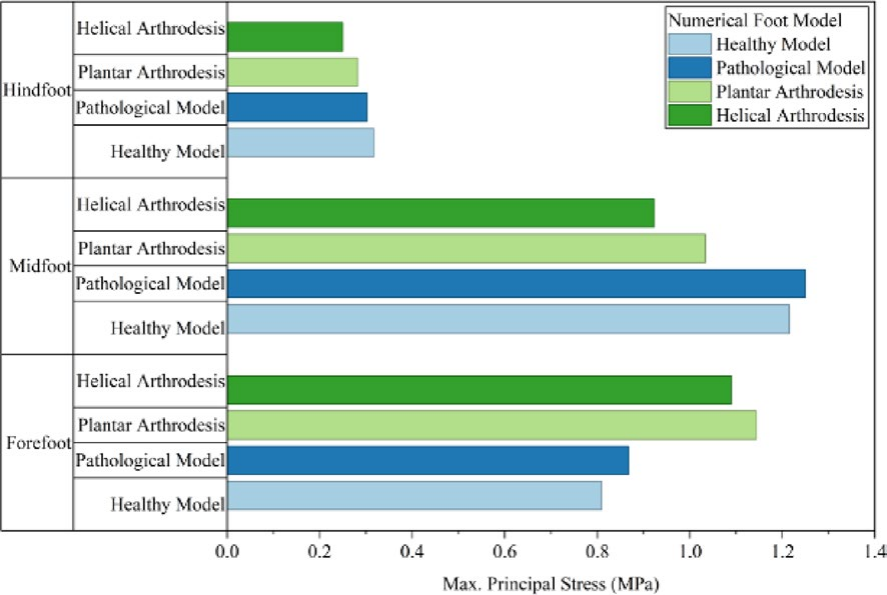
Principal stress values in different areas of the foot of the various models developed

Some structural parameters were evaluated to visualize and quantify the behavior of each foot model developed. The parameters considered are the maximum principal stress for bones, equivalent stress (von Mises) for the Lapidus plates, and total displacement for the CMJ bones. Although the models developed in this work are complex and composed of several bones and soft tissues, the study was primarily focused on the first metatarsocuneiform joint (CMJ), as the medial cuneiform and first metatarsal bones are the bone structures directly affected by the surgical procedure. Figures 17, 18, 19 and 20 show the comparison of the evaluated structural parameters for the different numerical models developed in this work.

### Electron microscopy results

Although no cracks or defects were observed superficially in any of the plates, the results obtained from the electron microscopy studies revealed interesting data, which are described below.

#### Plantar Lapidus plate

**Fig. 21:**
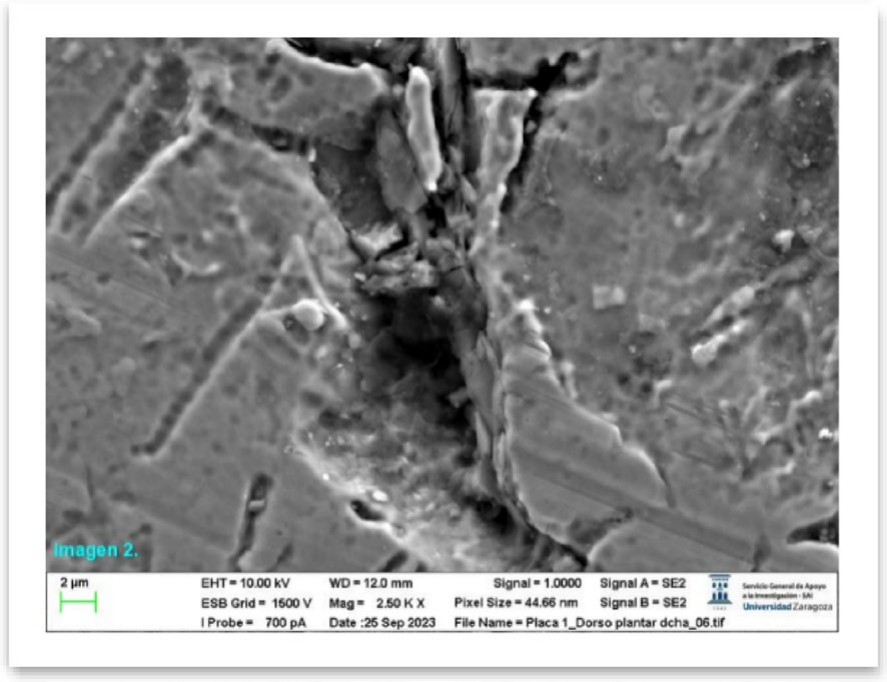
Electron microscopy evaluation of the plantar Lapidus plate. The image has been magnified 2500 times

During electron microscopy analysis, some cracks were seen in hole number 3 of the left group of the plantar Lapidus plate; in the central area of ​​the ventral face (between the commercial letters), non-conductive areas were evident, characterized by translucent whitish regions due to excessive electron charging and on the upper dorsal face of the plate, detachment of the smooth surface was observed, with large areas of non-electrical conduction and microperforations were visible, as shown in Fig. 21.

#### Helical Lapidus plate

**Fig. 22:**
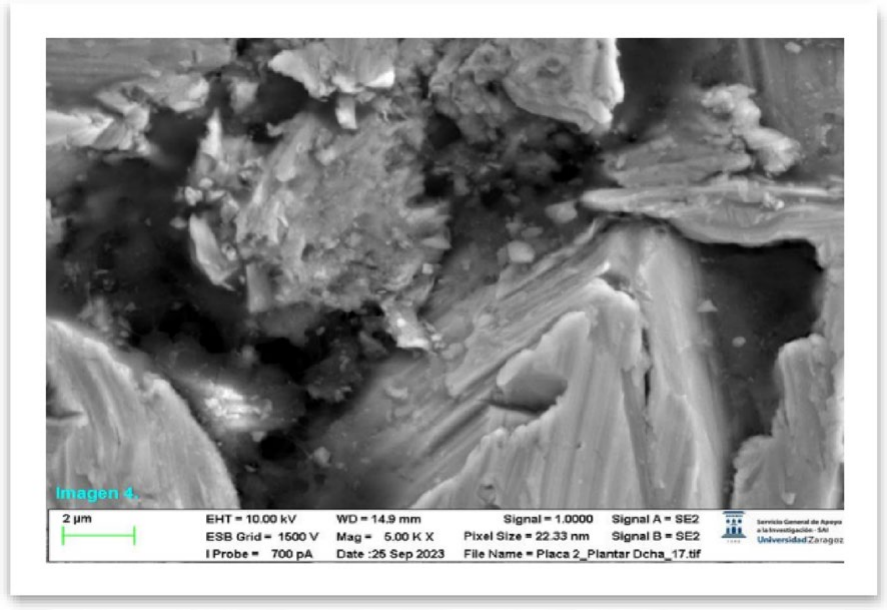
Electron microscopy evaluation of the helical Lapidus plate. The image has been magnified 5000 times

Regarding the helical Lapidus plate, the most notable features of the findings from its electron microscopy analysis are the multiple areas of erosion on the dorsal surface (see Fig. 22). To appreciate this, an image of the indicated area on the plate was captured at 5000× magnification. Initially, all the aforementioned details were considered as possible defects that the Lapidus plates may have suffered during the experimental tests. However, the defects found were attributed to manufacturing conditions, treatment before instrumentation with strain gauges, and manipulations to which the plates had been exposed, before or after the tests.

Given that the microscope can analyze areas (emission spectra) to detect traces of steel, the surface of the prosthetic component was examined using scanning electron microscopy (SEM) to characterize the morphology of the observed scratch. To determine the origin of the damage, energy-dispersive X-ray spectroscopy (EDS) was performed on the affected area. The EDS analysis revealed the presence of Fe, Cr, and Ni, characteristic of stainless steel. The detection of these elements indicates material transfer from a surgical instrument, supporting the hypothesis that the scratch was produced by mechanical contact during handling rather than by fatigue-related mechanisms. Furthermore, the absence of typical fatigue features (such as striations or crack initiation sites) in the SEM observations reinforces this interpretation.

### Results discussion

The most relevant findings that could be observed with the results obtained previously are the following:

- Experimentally, the helical plate demonstrates superiority in terms of the compressive force it supports during testing.
- The findings from electron microscopy did not suggest potential failures of the plate structures; however, more detailed patterns, such as possible very superficial cracks, were reported regarding the plantar plate following mechanical testing. These findings are attributed to the treatment of the plates during the strain gauge instrumentation.
- Helical plate arthrodesis reported higher strain values, occurring in the first metatarsal bone. On the other hand, the highest strain values in the plantar plate arthrodesis model occurred in the fifth metatarsal bone. According to the literature, postoperative findings indicate that the first metatarsal exhibits good tolerance to extension and that there is a significant correlation between hindfoot alignment and first metatarsal rotation [68]. Therefore, in this case, not only are the functional results of the foot not compromised, nor are the lower metatarsals overloaded, but the superior functionality of the helical plate contributes to improving the windlass mechanism and the hindfoot alignment, according to results reported by Grabielle Colo et al. [68]. Although both plates show a reasonable degree of hypermobility correction, the construction with a plantar plate is slightly stiffer.
- Arthrodesis with a helical plate reproduces the body load distribution with greater accuracy than the plantar plate arthrodesis, as it is closer to that shown in the healthy foot model.
- The stress in the cortical layer of the bones is practically similar between both arthrodesis models, with only a 1% difference closer to the healthy model, which is obtained in the model with the helical plate.
- The helical construct shows a greater reduction in the stress level of the trabecular layer than that demonstrated by the plantar construction.
- The helical plate experiences a higher level of stress than the plantar plate, reaching up to 45.23% of the material’s ultimate stress under the conditions established for the analysis.
- The plantar plate achieves a percentage of hypermobility correction that is 8% higher than that obtained with the helical plate.
- Both constructions generate increased stress in the forefoot compared to the healthy model. The most significant increase is observed in the plantar plate, showing a difference of up to 40% compared to a healthy foot. This represents a 6% increase over the increase observed with helical arthrodesis.
- The stresses in the midfoot and hindfoot are lower in the model with helical arthrodesis.

Despite efforts to develop a complete foot model, certain limitations were necessary to achieve acceptable results, avoiding the complexity associated with this member. One of them was the mechanical behavior of bones and tissues, which has been established as elastic, linear, and isotropic. And the second, the geometry of the peroneus longus tendon was not included, but only with the force that it exerts on the metatarsocuneiform joint.

In this work, the maximum percentage of strain reached by soft tissues was 8.9% (0.089 mm/mm). Under the analyzed conditions, it is possible to work by raising the hypothesis that the deformations are small under the conditions applied to the model, and it is not strictly necessary to use non-linear material models [58, 63, 69]; so that, the stress distributions found in the results will not show a significant variation if non linear material models were employe.However, if analysis conditions are modified and critical positions and loads are considered, leading the soft tissues to operate in the range of large deflection, non-linear material models must be used to avoid significantly inaccurate results.

It is also important to mention the limited availability of cadaveric specimens, which reduces the statistical power of this study. However, the consistency between the experimental and numerical analysis findings, the magnitudes of the observed structural parameters, and comparisons with the literature [6] support the validity of the findings and conclusions.

## Conclusions

Experimental compression tests revealed that the Lapidus plates sustained no apparent damage to their structure in any of the evaluated cases, even when the maximum load that both specimens were capable of supporting was reached. During these tests, it was also possible to verify that the specimens could support up to 3.9 times the average body weight of a person (70 kg), in the case of the plantar sample, which supported the least load. The foot implanted with a helical plate supported twice the aforementioned value.

The strain values found in the experimental tests suggest better functional performance of the helical Lapidus plate, as there is greater tolerance to extension by the first metatarsal compared to the lesser metatarsals.

The validation of the arthrodesis foot models also allowed for various observations. First, the comparison of the deformations obtained with both numerical models (plantar and helical) concerning the experimental evaluations showed a similar behavior, with a correlation of up to 98%. The above proves once again that the plantar Lapidus plate could lead to behavior that ultimately compromises the proper functioning of the foot, contrary to the model with a helical plate. Loading distribution across the foot was reproduced with a slightly higher accuracy percentage (95.83%) in the helical arthrodesis model compared to that reported in the literature. The plantar plate showed an accuracy rate of 94.53% compared to the literature. Although both models adequately validated the evaluated parameters, a slight superiority was observed, favoring the structural, functional, and biomechanical performance of the helical plate.

The evaluation of both arthrodesis foot models has shown that the insertion of Lapidus plates has a significant effect on the stress levels generated in the joint. The highest stresses are generated in the cortical layer of both bones (cuneiform and first metatarsal), with values ​​slightly higher than 2 MPa. Much lower stresses are found in the trabecular layer of the bones, with values ​​up to 0.21 MPa. However, it can be observed that, except for the cortical layer of the first metatarsal, stress levels tend to decrease during the pathology and even after the surgical procedure. This suggests that the insertion of Lapidus plates tends to concentrate most of their stress effects on the superficial layer of the first metatarsal bone. Although the stress values generated in this area do not reach critical levels under the conditions analyzed, it is essential to monitor this area after the procedure. Regarding the stresses generated in each Lapidus plate, it was observed that the most significant magnitude of this parameter occurred in the helical Lapidus plate. Stress values ​​of up to 79.16 MPa were reached there, six times greater than the stresses in the plantar Lapidus plate. However, the stresses generated in each bone were lower in the helical arthrodesis model. This suggests that the helical plate has a less significant effect on the joint in terms of stress.

The evaluation of the displacements occurring in the bones of the CMJ reveals a significant increase in movement levels in this area when the characteristic behavior of the pathology is reproduced (pathological foot model). Subsequently, the excess mobility is corrected up to 82.28% with the helical model, which shows a slightly greater difference compared to the movement measured in the healthy foot model. All the results comparisons between the two arthrodesis models demonstrated a slight biomechanical, structural, and functional superiority of the helical Lapidus plate.

One of the primary postoperative evaluations conducted on patients undergoing this surgical procedure is the recovery time and the return to walking with full body weight support. It is considered that the superiority shown by the helical plate (helical), although not significantly different from the functional, biomechanical, and structural capacities demonstrated by the plantar plate, can contribute to shorter recovery times for patients, and that the quality of gait after the procedure is also more suitable for them.

## Data Availability

All data that support the findings of this study are included within the article (and any supplementary files).

## Abbreviations

FCMJ: First cuneometatarsal joint
HV: Hallux valgus
CMJ: Cueneometatarsal joint
CPF: Central plantar fascia
LPF: Lateral plantar fascia
AH: Hallucis muscle
ADM: Abductor digiti minimi
MFDB: Flexor digitorum Brevis muscle
TFDB: Flexor digitorum Brevis tendon
EHL: Extensor hallucis longus
FDL: Flexor digitorum longus
FHL: Flexor hallucis longus
TA: Tibialis anterior
TP: Tibialis posterior
EDB: Extensor digitorum Brevis
